# Testing lifecourse theories characterising associations between maternal depression and offspring depression in emerging adulthood: the Avon Longitudinal Study of Parents and Children

**DOI:** 10.1111/jcpp.13699

**Published:** 2022-09-12

**Authors:** Rebecca E. Lacey, Dawid Gondek, Brooke J. Smith, Andrew D. A. C. Smith, Erin C. Dunn, Amanda Sacker

**Affiliations:** ^1^ Research Department of Epidemiology and Public Health University College London London UK; ^2^ Psychiatric and Neurodevelopmental Genetics Unit Center for Genomic Medicine Massachusetts General Hospital Boston MA USA; ^3^ Mathematics and Statistics Research Group University of the West of England Bristol UK; ^4^ Department of Psychiatry Harvard Medical School and the Massachusetts General Hospital Boston MA USA; ^5^ Stanley Center for Psychiatric Research The Broad Institute of Harvard and MIT Cambridge MA USA; ^6^ Center on the Developing Child at Harvard University Cambridge MA USA

**Keywords:** accumulation, ALSPAC, depression, depressive symptoms, lifecourse, sensitive periods

## Abstract

**Background:**

Maternal depression is a major determinant of offspring mental health. Yet, little is understood about how the duration and timing of maternal depression shapes youth risk for depressive symptoms, which if understood could inform when best to intervene. This study aimed to determine how the timing and duration of maternal depression was related to offspring depression in emerging adulthood, and if these associations varied by sex.

**Methods:**

We analysed data from the Avon Longitudinal Study of Parents and Children (a prenatal cohort in the Avon area of England, 1991–2003), *n* = 3,301. We applied the structured lifecourse modelling approach to maternal depression (assessed at 13 points from prenatal period to adolescence) and emerging adult depressive symptoms (age 21). Lifecourse models assessed were *accumulation* (sum of timepoints when maternal depression was reported), *sensitive periods* (each period assessed as one during which maternal depression has a stronger effect) and *instability* (frequent fluctuations in maternal depression).

**Results:**

Female adolescents (*n* = 2,132) had higher SMFQ scores (mean = 6.15, *SD* = 5.90) than males (*n* = 1,169, mean = 4.87, *SD* = 4.82). Maternal depression was most common in the infancy period (21.2% males; 21.4% females). For males, accumulation was the most appropriate lifecourse model; for each additional period of maternal depression, depressive symptoms in emerging adulthood increased by 0.11 (95% CI: 0.07, 0.15, one‐sided *p* value ≤ .001). For females, exposure to maternal depression was associated with increasing depressive symptoms in emerging adulthood, with the largest effect in mid‐childhood (increase of 0.27 units, 95% CI 0.03–0.50, *p* = .015 for difference between mid‐childhood and other time‐periods) and a smaller, equal effect at all other time‐periods (increase of 0.07 units per time‐period, 95% CI: 0.03–0.12, *p* = .002).

**Conclusions:**

This study highlights the importance of ongoing maternal depression for the development of depression in offspring through to emerging adulthood. Because long‐term exposure to maternal depression was particularly important, early interventions are warranted.

## Introduction

Maternal depression has been identified as a relevant risk factor for the mental health of children (McLaughlin et al., 2012; Sanger, Iles, Andrew, & Ramchandani, 2015). Analysis of the Avon Longitudinal Study of Parents and Children (ALSPAC) found that for each increasing standard deviation on the Edinburgh Postnatal Depression Scale, maternal depression in pregnancy was associated with 1.3 times increased odds of offspring depression nearly two decades later (Pearson et al., 2013). Similarly, a systematic review demonstrated that maternal postnatal depression was associated with mental health difficulties of children in adolescence, but noted future research is needed to research the timing and duration of experience of maternal depression (Sanger et al., 2015).

Indeed, most previous research has focused on pre‐ or postnatal depression alone, ignoring the continuation of maternal depression into later stages of childhood, adolescence and into early adulthood. One previous study found that mothers who had postnatal depression were substantially more likely to suffer subsequent episodes of depression and that in such situations of ongoing maternal depression, their adolescent offspring were six times more likely to have depression (Halligan, Murray, Martins, & Cooper, 2007). Similarly, a smaller scale study (151 mother–child pairs) found that every 16‐year‐old with depression had been exposed to maternal depression at some point earlier in their lifecourse (Pawlby, Hay, Sharp, Waters, & O'Keane, 2009), with the effect of maternal depression during pregnancy being most pronounced, although repeated exposure during early life was also important. These findings emphasise the importance of considering the developmental timing and duration of maternal depressive episodes on offspring mental health.

Further, the association between maternal depression and offspring depression could differ by sex, as females have been shown to be more vulnerable to family problems – including maternal depression – than males, due to being socialised to be more family‐oriented (Sheeber, Davis, & Hops, 2004). However, there is some inconsistency in the findings of studies considering sex differences in maternal depression on offspring mental health. For example, analyses (Mensah & Kiernan, 2010) in ALSPAC found that males aged 18 were more likely to have depression if their mothers had experienced both prenatal and postnatal depression and that these sex differences emerged during adolescence (Quarini et al., 2016). Conversely, the association between postnatal depression and offspring anxiety and depressive disorders was stronger in females than in males (Halligan et al., 2007), although this finding was based on a small‐scale longitudinal study of 94 mother–child pairs. Duration of exposure to maternal depression is also likely to be important. Previous work has shown that long‐term maternal depression was associated with more internalising problems among females (Foster et al., 2007). Hence, while there is some inconsistency in findings, evidence from previous studies seems to tentatively point towards females being more strongly influenced by experiences like maternal depression but sex‐stratified associations warrant more attention using larger and more generalisable longitudinal studies.

### The importance of the lifecourse approach

Lifecourse theories explain how the timing, changes in, and duration of life experiences, such as maternal depression, can influence lifelong mental health and therefore suggest different avenues for intervention (Ben Shlomo & Kuh, 2002). Frequently studied lifecourse models are *accumulation of risk* and *sensitive periods in development* (Dunn, Soare, et al., 2018). Within the context of our study, *accumulation* represents the number of occasions a child was exposed to maternal depression during childhood and adolescence, regardless of the timing of their occurrence. Evidence supporting the accumulation model could point researchers towards intervening early in development to prevent adverse life experiences from accruing further. A *sensitive period* is a window of time during development when exposure to maternal depression may be most strongly related to increased risk of offspring depression and thus when interventions could have most impact. We hypothesise that a sensitive period exists in infancy or early childhood when the child is most dependent on the mother (Naicker, Wickham, & Colman, 2012). More recently, a *mobility* model has been proposed whereby the change between adjacent time‐periods is important. Typically, mobility has been operationalised with respect to socioeconomic position (Liu et al., 2022; Mishra et al., 2009). We re‐label this mobility model as *instability* here, because for maternal depression, the lack of stability and predictability may be most detrimental for youth. Depression is a condition in which there can be frequent fluctuations in severity. Parents who recover from a depressive episode may still have sub‐threshold levels of symptoms, which may have knock‐on consequences for parenting and result in children who have frequent experiences of parental depression (Beardslee, Gladstone, & O'Connor, 2011). There is evidence that the recency of episodes can impact on young people's depression. Further, evidence from qualitative studies of young people with parents with depression finds that instability or unpredictability of parental moods and depressive symptoms affects family functioning and their own wellbeing (Trondsen, 2012). Disruptions to parenting caused by instability of parental depression is a key mechanism linking parental depression to young people's mental health problems (Smith, 2004). Interventions based on the instability model make the impetus on early and continuous intervention.

### Testing lifecourse models

Researchers are increasingly using systematic approaches to identify the lifecourse models reflecting disease aetiology to inform the optimal timing of interventions. Systematic approaches require the analyst to test pre‐specified hypotheses, which clearly define their models of interest (Hardy & Tilling, 2016). Systematic approaches also recognise that some (or even all) of the pre‐specified hypotheses could potentially work in combination. A recent approach – the Structured Life Course Modelling Approach (SLCMA; pronounced “slick‐mah”) – has been proposed to select the best‐fitting models based on least angle regression (LARS; Smith et al., 2015). The SLCMA identifies the lifecourse model most supported by observed data, enabling researchers to go beyond simple exposed/unexposed analyses. Furthermore, the SLCMA allows for the discovery of lifecourse models working in combination, which could provide insights into disease mechanisms and opportunities to intervene. To date, the SLCMA has been applied to studies on multiple early‐life adversities (including maternal psychopathology) and epigenetic ageing (Marini et al., 2020), DNA methylation (Dunn et al., 2019; Liu et al., 2022), and childhood psychopathology outcomes (Dunn, Soare, et al., 2018; Smith, Smith, & Dunn, 2022) in ALSPAC. In these studies, sensitive periods were most often found to be more appropriate than accumulation. These studies mainly focused on an exposure window of early childhood rather than across the whole early lifecourse (from pregnancy through to early adulthood).

In the present study, we extend prior research to apply SLCMA to maternal depression. We investigate three lifecourse models – accumulation, sensitive periods and instability – across early life (from the prenatal period through adolescence) to explore their importance for depressive symptoms in emerging adulthood in a large‐scale prospective longitudinal study. Given the findings of previous literature on maternal depression and that SLCMA studies on other early life adversities consistently identified the importance of sensitive periods, we hypothesised that a sensitive period in infancy or early childhood would fit best. In our study, we are able to extend the findings of prior studies by including potential sensitive periods right through to adolescence – one of the first studies to do so. Further, based on prior literature, we hypothesised that associations would be stronger for females.

## Methods

### Study design

Data came from ALSPAC – a prenatal cohort from the Avon region of South‐West England. ALSPAC initially recruited 14,541 pregnant mothers with estimated due dates between April 1991 and December 1992 (Boyd et al., 2012; Fraser et al., 2013; Northstone et al., 2019). When the children were aged 7, an additional 913 children with the same eligible dates of birth as the original cohort members were enrolled, resulting in 15,454 children, of whom 14,901 were alive at 1 year of age. There have been many waves of data collection from the prenatal period through adulthood, collecting measures of social, economic, health and developmental aspects. Cohort offspring are now completing their age 30 assessment. Further details of the design of ALSPAC can be found elsewhere (Boyd et al., 2012; Fraser et al., 2013). Ethical approval was obtained from the ALSPAC Ethics and Law Committee and the Local Research Ethics Committee. Informed consent was provided from all participants or their carers for each data collection. The study website contains details of all the data that are available through a fully searchable data dictionary and variable search tool: http://www.bristol.ac.uk/alspac/researchers/our‐data/.

### Maternal depression

Maternal depression was assessed 13 times from pregnancy through child age 19 (Table S1). On nine occasions ALSPAC investigators administered the 10‐item Edinburgh Postnatal Depression Scale (EPDS) to mothers. The specific questions are detailed in Table S2. Scores of 13 or more had high specificity (96%) and sensitivity (81%) in predicting major depressive disorder (Eberhard‐Gran, Eskild, Tambs, Opjordsmoen, & Samuelsen, 2001). The EPDS has also been validated for use in non‐postnatal women, with acceptable levels of sensitivity (79%) and specificity (85%; Cox, Chapman, Murray, & Jones, 1996). On the other four occasions, mothers were asked whether they had depression and further whether they had consulted a doctor within a certain time‐period (recollection period varied across data sweeps; Table S1). An affirmative response for both questions indicated likely the presence of maternal depression. These 13 binary indicator variables were used to construct the variables describing each lifecourse model – accumulation of risk, sensitive periods and instability. We identified six potential sensitive periods for maternal depression in the life of the cohort child [prenatal (18–32 weeks' gestation), infancy (0–2 years), early childhood (3–5 years), middle childhood (6–8 years), late childhood (9–12 years), adolescence (age 19)], based on the developmental theory (Levesque, 2011). Details on how each of the accumulation, sensitive period and instability variables were created are detailed in Table 1.

**Table 1:** Description of the tested lifecourse models investigated through the SLCMA

Lifecourse model	Estimate	Effect size
Sensitive period	The proportion of explained variability in emerging adult mental health by a period at which exposure to maternal depression had a particularly strong association with emerging adult mental health. Six life periods were distinguished: Period 1: prenatal (age 18–32 weeks gestation) Period 2: infancy (age 0–2 years) Period 3: early childhood (age 3–5 years) Period 4: middle childhood (age 6–8 years) Period 5: late childhood (age 9–12 years) Period 6: adolescence (age 19 years)	*R* ^2^ explained by five variables testing the model: depressed_period1 = exposed (1) vs. unexposed (0) depressed_period2 = exposed (1) vs. unexposed (0) depressed_period3 = exposed (1) vs. unexposed (0) depressed_period4 = exposed (1) vs. unexposed (0) depressed_period5 = exposed (1) vs. unexposed (0) depressed_period6 = exposed (1) vs. unexposed (0)
Accumulation	The proportion of explained variability in emerging adult mental health by the number of times a participant was exposed to maternal depression throughout distinct periods of early life	*R* ^2^ explained by 1 variable testing the model: depressed_accumulation = depressed_period1 + depressed_period2 + depressed_period3 + depressed_period4 + depressed_period5 + depressed_period6
Instability	The proportion of explained variability in emerging adult mental health by the number of times a participant was exposed to change in maternal depressive state between adjacent life periods (i.e. from depressed to non‐depressed or vice versa)	*R* ^2^ explained by one variable testing the model: depressed_instability = (depressed_period1 ≠ depressed_period2) + (depressed_period2 ≠ depressed_period3) + (depressed_period3 ≠ depressed_period4) + (depressed_period4 ≠ depressed_period5) + (depressed_period5 ≠ depressed_period6)

### Offspring depressive symptoms

Emerging adult depressive symptoms were measured with the Short Mood and Feelings Questionnaire (SMFQ; Messer, Angold, Costello, van Kämmen, & Stouthamer‐Loeber, 1996), which was administered via postal/email questionnaire and completed by cohort members at age 21. The SMFQ consists of 13 items that measure depressive symptoms in the last 2 weeks (see Table S3 for more details). Summed scores ranged from 0 to 26, with higher scores indicating more depressive symptoms. The SMFQ at age 21 has good reliability: internal consistency assessed by Cronbach's alpha >0.90.

### Covariates

Potential confounding variables were based on those used in prior studies (Dunn, Soare, et al., 2018; Pearson et al., 2013) were measured from birth: *child's sex* (female or male), *pregnancy size* (single or multiple), *maternal partnership status* (married, single, widowed/separated/divorced), *maternal education* (vocational, Certificate of Secondary Education, O‐levels, A‐levels or degree), *maternal social class during pregnancy* based on occupation (professional/managerial and technical/skilled non‐manual/skilled manual/partly skilled/unskilled), and *experience of poverty during pregnancy* (being homeless and/or having difficulty affording food, heating or accommodation).

### Statistical analysis

#### Multiple imputation

Missing information was replaced using multiple imputation by chained equations to minimise the bias due to attrition and non‐response. Totally 20 imputed datasets were created. Further information is given in Appendix S1.

#### Testing lifecourse models

Because the SLCMA cannot detect the best lifecourse hypothesis in the presence of strongly correlated exposures (*r* > .9; Smith et al., 2015), correlations were estimated to check bivariate associations between period‐specific exposures to maternal depression. We implemented the SLCMA to assess the fit of the theorised models. Following the approach recommended by Smith et al. (2015), we used LARS (Efron, Hastie, Johnstone, & Tibshirani, 2004) to identify the theoretical lifecourse model or models that explained the most variability in late adolescent depression. Variables describing each theoretical lifecourse model (accumulation of risk, sensitive period, instability; see Table 1) were entered into LARS, separately for males and females as sex differences were shown in previous ALSPAC analyses (Dunn, Soare, et al., 2018). We adjusted for covariates by regression exposure variables and age 21 SMFQ score on covariates and using regression residuals for the subsequent analysis. This approach has been recommended for use with SLCMA (Zhu et al., 2019). The number of key variables included in the final model was determined using an elbow plot. Once the best‐fitting lifecourse hypothesis was identified, we implemented selective inference, a post‐selection inference method, to estimate effect sizes, confidence intervals, and *p* values. Selective inference has been recommended due to its ability to control family‐wise error rate and provide optimal statistical power as well as confidence interval coverage (Tibshirani et al., 2019; Tibshirani, Taylor, Lockhart, & Tibshirani, 2016; Zhu et al., 2019).

## Results

### Descriptive and bivariate statistics

The analytic sample was predominantly white (96%), including 35% males and 65% females. Females had a higher mean SMFQ score than males (mean = 6.15, standard deviation, *SD* = 5.90 vs. mean = 4.87, *SD* = 4.82; see Table S4 for more details). The prevalence of maternal depression was highest during the infancy period (21.2% males and 21.4% females were exposed) and lowest when their offspring were aged 3–5 years (15.8% males and 16.8% females were exposed). Table S5 shows the prevalence of maternal depression at every time point. Most children were born to married mothers (86.2% males; 83.2% females). The modal maternal education category was a university degree qualification. About 10% of cohort mothers were in poverty during pregnancy. Most children had mothers who were in skilled non‐manual or managerial, technical occupational – social classes during pregnancy.

**Figure 1:**
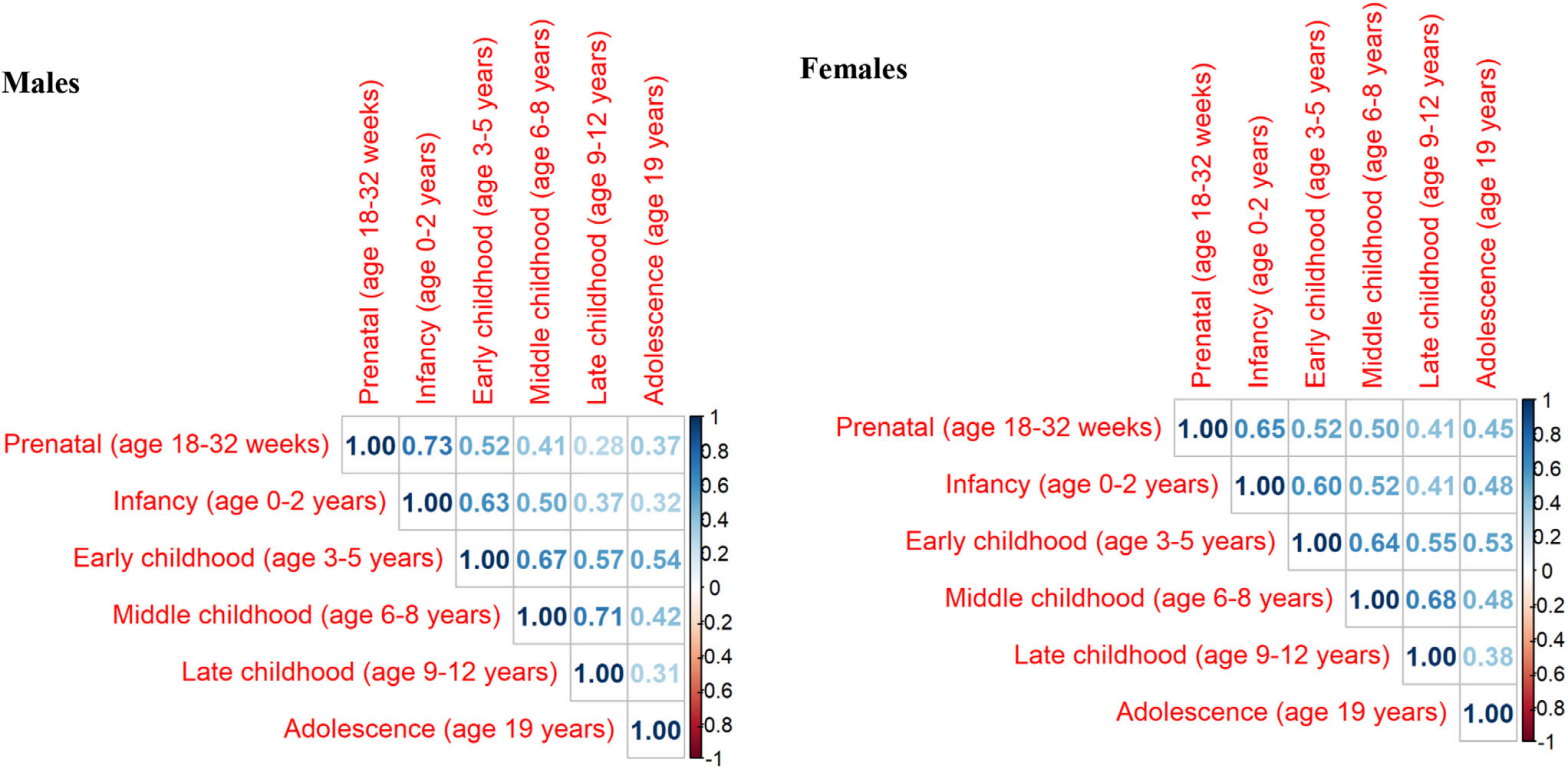
Tetrachoric correlations between maternal depression at different offspring lifecourse stages – stratified by sex

Tetrachoric correlation between exposure to maternal depression at different life periods was moderate to high (*r* = .28–.73; Figure 1). As expected, adjacent lifecourse periods had typically stronger correlation than more distal lifecourse periods.

### SLCMA findings

**Figure 2:**
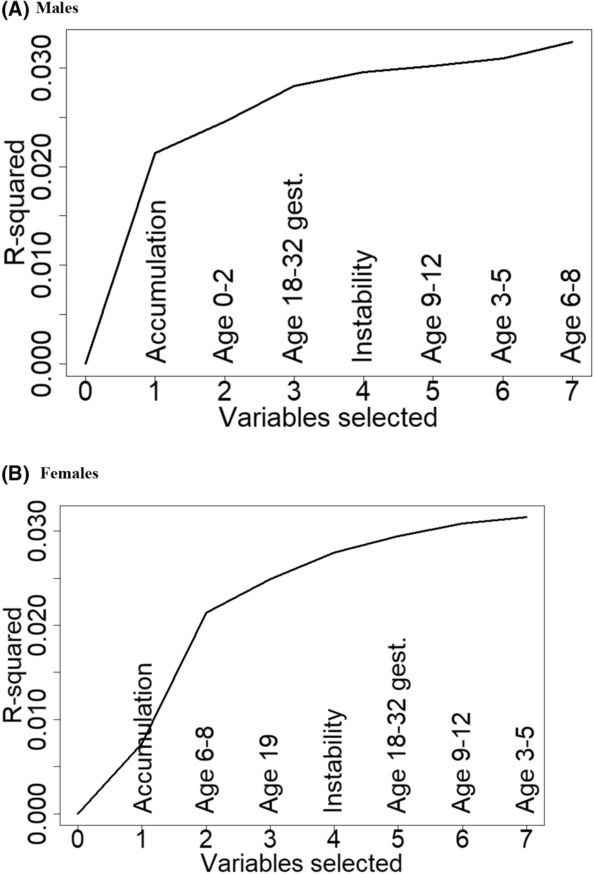
Elbow plots for Least Angle Regression (LARS) variable selection testing lifecourse models of exposure to maternal depression – stratified by sex. The elbow plot shows the *R* ^2^ explained by a model after adding each additional lifecourse model variable. The first peak in the plot indicates the optimal number of variables. Additional variables added beyond this point bring diminishing returns in *R* ^2^ and complicate the interpretation of the model

**Figure 3:**
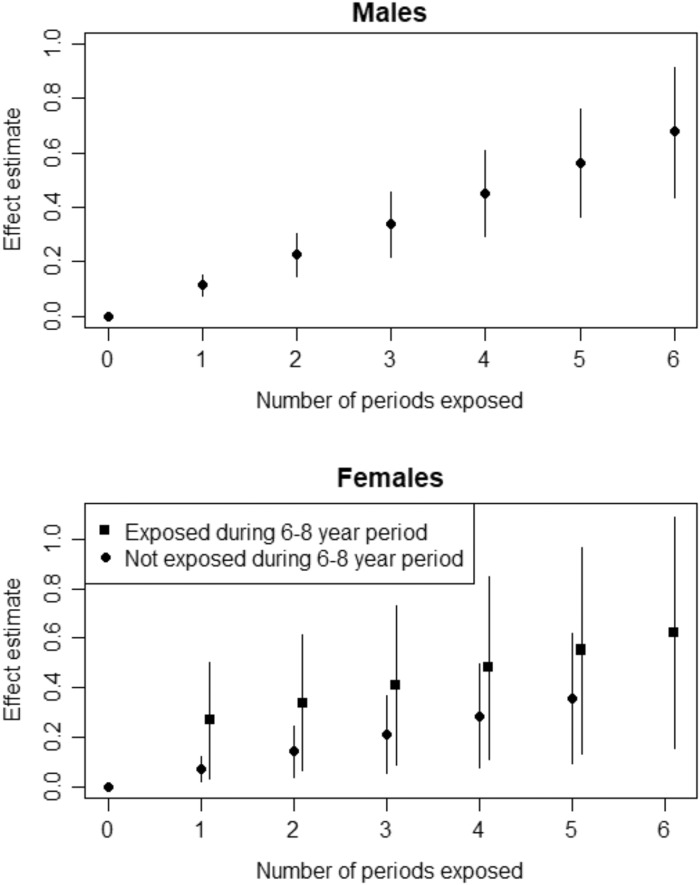
Effect estimates (95% CIs) for selected lifecourse hypotheses for males and females. The effect estimates on which these figures are based are given in Table S6. For males, increasing occasions of maternal depression are associated with increasing SMFQ scores at age 21; for females, increasing occasions of maternal depression are associated with increasing SMFQ scores, with the largest effect seen for those who experienced maternal depression between ages 6–8 years

For males, accumulation of maternal depression across the early lifecourse was best supported in our data (Figure 2). For females, accumulation plus a sensitive period in mid‐childhood (6–8 years) was best supported. In the case of males, every additional time‐period of exposure to maternal depression was associated, on average, with 0.11 unit higher depressive symptoms in emerging adulthood (95% CI: 0.07, 0.15, *p* < .001; Figure 3, Table S6). For females, exposure to maternal depression was associated with increasing depressive symptoms in emerging adulthood, with the largest effect in mid‐childhood (increase of 0.27 units, 95% CI: 0.03–0.50, *p* = .015 for difference between mid‐childhood and other time‐periods) and a smaller, equal effect at all other time‐periods (increase of 0.07 units per time‐period, 95% CI: 0.03–0.12, *p* = .002).

## Discussion

Using prospective longitudinal data, we found that emerging adulthood depressive symptoms were best explained by the accumulation of exposure to maternal depression and not the infancy sensitive period model, as we had hypothesised. For females, we also identified a sensitive period in mid‐childhood when maternal depression had a stronger association than other time periods. While the *R* ^2^ values might appear small, they are in line with what we would expect in a population (rather than clinical) cohort when analysing a single exposure and are consistent with other SLCMA analyses (Dunn et al., 2019).

Few studies considered the prevalence of maternal depression across time and there is considerable variability in the prevalence of maternal depression reported. For instance, a systematic review of studies estimating the prevalence of maternal postnatal depression reported prevalences of between 8.5%–23.0% in studies across Europe – most of which used the EPDS, as in this study (Arifin, Cheyne, & Maxwell, 2018). The prevalence of postnatal depression in our study was 21%, hence was consistent with prior studies included in this review. Our estimates of prevalence across other age periods were consistent with other studies using ALSPAC, for example, Paul and Pearson (2020), although are lower than those shown in the UK Millennium Cohort Study, for example (Hope, Deighton, Micali, & Law, 2019), which is a more representative cohort.

Our findings align with many studies demonstrating the importance of accumulation of exposures in early life. Relating specifically to maternal depression, analysis of a Brazilian longitudinal study (Pires et al., 2020) showed that accumulation of exposure to maternal depression was associated with more child mental health problems. Similarly, follow‐up of the ALSPAC cohort found that accumulation of maternal depression status was associated with lower child cognitive ability at age 8 (Evans et al., 2012). Analyses of a longitudinal study in Adelaide, Australia showed that accumulation of maternal depression up to age 9 was associated with more child internalising problems (Giles et al., 2011). Analyses of the Australian Longitudinal Study on Women's Health (Moss, Dobson, & Mishra, 2020) explored the best‐fitting lifecourse models for the association between maternal depression and child socioemotional behaviours (indicated by the Strengths and Difficulties Questionnaire, SDQ), finding that accumulation was the best‐fitting model; children exposed to more periods of maternal depression had higher SDQ scores. Our findings are consistent with this work but demonstrate this relationship extends across the early life course into emerging adulthood.

The finding of a mid‐childhood sensitive period for females was surprising given previous literature indicating the importance of infancy as a sensitive period for adult depression, following exposure to child maltreatment (Dunn, Nishimi, Powers, & Bradley, 2016). However, few studies have explored sensitive periods across the early lifecourse, as in the present study, instead focussing on the effect of maternal depression during the antenatal period alone. Two longer term studies also found that exposure to maternal psychopathology in mid‐childhood was associated with adverse childhood outcomes; Dunn, Nishimi, Gomez, Powers, and Bradley (2018) showed that exposure to interpersonal stressors, such as family disruption and parental mental illness in mid‐childhood (6–10 years) were associated with higher emotional dysregulation in adulthood in a retrospective study of health service seeking adults in Atlanta, Georgia.

Sex differences were observed. We saw that accumulation and a sensitive period in mid‐childhood were operating simultaneously in females, but only accumulation was present in males. This disparity in lifecourse models identified occurred despite females and males being equally exposed to maternal depression (i.e. having similar prevalence), at least on a reporting level. It is possible that females with depressed mothers provide more emotional support to their mothers than males, creating emotional strain and stress for females (Sheeber et al., 2004), although this information was not captured in our study. The work by Marini et al. (2020) in ALSPAC which applied SLCMA to relationships between several adversities and a marker of epigenetic ageing found that a sensitive period at age 6 for exposure to maternal psychopathology and epigenetic ageing for females only. Further research is needed to determine why this sex differences might emerge, particularly in this age period. Potential avenues might include exploring aspects of parenting and social (e.g. peer) support, which might differ for males and females.

### Strengths and limitations

This study is not without limitations. First, there was inconsistency in the measures of maternal depression across waves; the EPDS was unavailable at all 13 waves, requiring us to also use self‐reported seeking of medical advice for depression. It is possible that we are underestimating the prevalence of maternal depression at some waves. Further, due to sparsity in data collection, we were only able to include one wave of data in our “adolescent” lifecourse period, whereas other lifecourse periods combined information from at least two waves. This may have resulted in an under‐detection of maternal depression during adolescence. Also, our sample contained more females than males, a difference driven by reporting of the SMFQ at age 21, the only exclusion criteria for our study. The higher retention of female compared with male participants is common in many longitudinal studies (Teague et al., 2018). Males in our study might be less representative than females and the analyses for males may have lower statistical power. Further, missing information on the SMFQ at age 21 was the main source of missing information in our study, followed by missing information on maternal depression (Table S4). We were also unable to consider time‐varying confounders in our analyses, focusing only on covariates measured mainly at birth, as the SLCMA has not yet been adapted for such use. However, most covariates, for example, maternal education, did not change substantially over time. Similarly, maternal and child depressive symptoms may be bidirectional and this warrants further investigation with frequent and repeated measures of maternal and child depression over time, which we did not have here, could further elucidate these relationships. Also, we focused on one aspect of the family environment – maternal depression. Maternal depression may cluster with other adversities in a complex, time‐varying manner. As such, associations maybe confounded by other adverse experiences occurring during early life. Relatedly, we were also unable to look at paternal depression without substantially restricting our analyses to stable, two‐parent families.

Despite these limitations, our study has several strengths. First, we used data from a large, prospective longitudinal study. Many of the previous studies on maternal depression have been unable to take a long‐term view across the entire lifecourse period to explore lifecourse models that explain the association between mother and child depression. Second, we applied the SLCMA, enabling a rigorous examination of lifecourse models through to emerging adulthood. Third, we addressed missing data using multiple imputation to reduce bias. Finally, some of our maternal depression measures were reported by two respondents (mother and mother's partner), hence circumventing responder bias found in many longitudinal studies.

## Conclusion

This study highlights the importance of maternal depression for the development of depressive symptoms of children in emerging adulthood, emphasising here the effect that long‐term exposure to maternal depression might have. In many high‐income countries, there is a substantial focus on supporting maternal mental health during pregnancy and perinatally. Our findings suggest that this support should be maintained for longer, at least through childhood. Regular screening or mental health check‐ins are warranted to identify and effectively treat maternal mental health problems at an early stage to prevent the long‐term effect on families. Finally, given our findings on the importance of accumulation of maternal depression, more research is needed to understand the predictors of persistent maternal depression. Some studies exist in this area although they have typically only considered maternal depression over a short period (Horwitz, Briggs‐Gowan, Storfer‐Isser, & Carter, 2007; Yonkers et al., 2001). This information has the potential to inform interventions and ultimately improve mental health across generations.

## Supporting information


**Table S1.** Description of maternal depression measures in the ALSPAC study, assessed at 13 time points.
**Table S2.** Edinburgh Postnatal Depression scale items.
**Table S3.** Short Mood and Feelings Questionnaire items.
**Table S4.** Distribution of the study variables stratified by sex and missingness.
**Table S5.** Prevalence of maternal depression at individual time points.
**Table S6.** Effect estimates (95% CIs) for selected lifecourse hypotheses.
**Appendix S1.** Further information on multiple imputation.

## Data Availability

Data from ALSPAC are available via a managed open access system. Further information can be obtained here: http://www.bristol.ac.uk/alspac/researchers/access/.Key points
The mental health of parents is important for the development of mental ill‐health of children but a better understanding of exposure to maternal depression over time is needed to inform interventions. We set out to assess how the timing and accumulation of maternal depression shapes children's risk of depressive symptoms in emerging adulthood. Using prospective, longitudinal data we found that accumulation of maternal depression experience was the best‐fitting lifecourse model for males. For females, a sensitive period in mid‐childhood was identified in addition to the accumulation of maternal depression across early life. The findings point to the importance of identifying and treating maternal depression at an early stage to prevent long‐term effects on both the mother and child.
