# Supplementary material for: Testing lifecourse theories characterising associations between maternal depression and offspring depression in emerging adulthood: the Avon Longitudinal Study of Parents and Children
Source: J Child Psychol Psychiatry. 2022 Sep 12;64(8):1149–58. doi: 10.1111/jcpp.13699 (PMC10008452; doi:10.1111/jcpp.13699)
Supplement: Supplementary file 1 — Table S1. Description of maternal depression measures in the ALSPAC study, assessed at 13 time points. Table S2. Edinburgh Postnatal Depression scale items. Table S3. Short Mood and Feelings Questionnaire items. Table S4. Distribution of the study variables stratified by sex and missingness. Table S5. Prevalence of maternal depression at individual time points. Table S6. Effect estimates (95% CIs) for selected lifecourse hypotheses. Appendix S1. Further information on multiple imputation. [file JCPP-64-1149-s001.docx]

**Supporting Information**

| **Table S1.** Description of maternal depression measures in the ALSPAC study, assessed at 13 time points | | |
| --- | --- | --- |
| Age of child | Measure of depression | Coding of the variable |
| 18 weeks gestation | Edinburgh Postnatal Depression Scale | Depressed (1): score≥13  Not depressed (0): score<13 |
| 32 weeks gestation | Edinburgh Postnatal Depression Scale | Same as above for EPDS |
| 8 weeks | Edinburgh Postnatal Depression Scale | Same as above for EPDS |
| 8 months | Edinburgh Postnatal Depression Scale | Same as above for EPDS |
| 1 year and 9 months | Edinburgh Postnatal Depression Scale | Same as above for EPDS |
| 2 years and 9 months | Edinburgh Postnatal Depression Scale | Same as above for EPDS |
| 3 years and 11 months | “Have you had any of the following in the past year?” (depression) | Depressed (1): Yes and consulted doctor  Not depressed (0): Yes but did not consult doctor/ No |
| 5 years and 1 month | Edinburgh Postnatal Depression Scale | Same as above for EPDS |
| 6 years and 1 month | “Have you had (or continued to have) any of the following since your study child’s 5^th^ birthday” (depression) | Depressed (1): Yes and consulted doctor  Not depressed (0): Yes but did not consult doctor/ No |
| 8 years and 1 month | Edinburgh Postnatal Depression Scale | Same as above for EPDS |
| 9 years 1 month | “Have you had any of the following in the last 3 years (since your study child’s 6th birthday)?” (depression) | Depressed (1): Yes and consulted doctor  Not depressed (0): Yes but did not consult doctor/ No |
| 12 years 1 month | “Have you had any of the following in the last 2 years (since your study child’s 10th birthday)? (depression) | Depressed (1): Yes and consulted doctor  Not depressed (0): Yes but did not consult doctor/ No |
| 19 years | Edinburgh Postnatal Depression Scale | Same as above for EPDS |

EPDS = Edinburgh Postnatal Depression Scale

**Table S2.** Edinburgh Postnatal Depression scale items

| **Item** | **Response options and coding** | | | |
| --- | --- | --- | --- | --- |
| I have been able to laugh and see the funny side of things | As much as I always could (0) | Not quite so much now (1) | Definitely not so much now (2) | Not at all  (3) |
| I have looked forward with enjoyment to things | As much as I ever did  (0) | Rather less than used to (1) | Definitely less than I used to  (2) | Hardly at all  (3) |
| I have blamed myself unnecessarily when things go wrong | Yes, most of the time  (0) | Yes, some of the time  (1) | Not very often  (2) | No never  (3) |
| I have been anxious or worried for no good reason | No, not at all  (0) | Hardly ever  (1) | Yes, sometimes  (2) | Yes, often  (3) |
| I have felt scared or panicky for no very good reason | Yes, quite a lot  (3) | Yes, sometimes (2) | No, not much  (1) | No, not at all  (0) |
| Things have been getting on top of me | Yes, most of the time  (3) | Yes, sometimes  (2) | No, hardly ever  (1) | No, not at all  (0) |
| I have been so unhappy that I have had difficulty sleeping | Yes, most of the time  (3) | Yes, sometimes  (2) | Not very often  (1) | No, not at all  (0) |
| I have felt sad or miserable | Yes, most of the time  (3) | Yes, quite often  (2) | Not very often  (1) | No, not at all  (0) |
| I have been so unhappy that I have been crying | Yes, most of the time  (3) | Yes, quite often  (2) | Only occasionally  (1) | No, never  (0) |
| The thought of harming myself has occurred to me | Yes, quite often  (3) | Sometimes  (2) | Hardly ever  (1) | Never  (0) |

NB all questions ask about feelings in the past week

**Table S3.** Short Mood and Feelings Questionnaire items

| **Item** | **Response options and coding** | | |
| --- | --- | --- | --- |
| I felt miserable or unhappy | Not true  (0) | Sometimes  (1) | True  (2) |
| I didn’t enjoy anything at all | Not true  (0) | Sometimes  (1) | True  (2) |
| I felt so tired I just sat around and did nothing | Not true  (0) | Sometimes  (1) | True  (2) |
| I was very restless | Not true  (0) | Sometimes  (1) | True  (2) |
| I felt I was no good anymore | Not true  (0) | Sometimes  (1) | True  (2) |
| I cried a lot | Not true  (0) | Sometimes  (1) | True  (2) |
| I found it hard to think properly or concentrate | Not true  (0) | Sometimes  (1) | True  (2) |
| I hated myself | Not true  (0) | Sometimes  (1) | True  (2) |
| I was a bad person | Not true  (0) | Sometimes  (1) | True  (2) |
| I felt lonely | Not true  (0) | Sometimes  (1) | True  (2) |
| I thought nobody really loved me | Not true  (0) | Sometimes  (1) | True  (2) |
| I thought I could never be as good as others | Not true  (0) | Sometimes  (1) | True  (2) |
| I did everything wrong | Not true  (0) | Sometimes  (1) | True  (2) |

NB all questions ask about feelings in the past two weeks

**Appendix S1**. Further information on multiple imputation

In line with recommendations (Sterne et al, 2009; White et al, 2011), the imputation model included all the variables from the analyses, as well as interaction terms between adjacent periods (e.g., infancy – age 0-2 years*early childhood – age 3-5 years), ensuring that the relationship between the variables of interest was preserved. Multiple imputation assumes that information is missing at random (MAR), which implies that systematic differences between the missing and observed values can be explained by observed data (Hughes et al, 2019). Hence, we also enriched the imputation model and further maximised the plausibility of the MAR assumption by including auxiliary variables (smoking and drinking during pregnancy). These variables were not part of the substantive model of interest, but they were associated with missingness and/or adolescent depression. To reduce noise in the estimation of effect estimates, the outcome was not imputed (White et al, 2011), hence the final analytic sample comprised participants with complete information on the SMFQ at age 21 (n=3,301). We created 20 imputed datasets (Azur et al, 2011) with the *mice* package in R (Buuren and Groothuis-Oudshoorn, 2011). We averaged the covariance structure across the 20 multiply imputed datasets to obtain a single dataset for analysis (Wood, White and Royston, 2008).

| **Table S4.** Distribution of the study variables stratified by sex and missingness | | | | | | | | |
| --- | --- | --- | --- | --- | --- | --- | --- | --- |
|  | **Males** | | | | | **Females** | | |
| **Variable** | **Mean (SD) MI** | | **N (Missing)** | | **Mean (SD) CC** | **Mean (SD) MI** | **N (Missing)** | **Mean (SD) CC** |
| SMFQ | 4.87 (4.82) | | 1,169 (0) | | 4.87 (4.82) | 6.15 (5.90) | 2,132 (0) | 6.15 (5.90) |
| Mother's age | 30.0 (4.36) | | 1,090 (79) | | 30.00 (4.36) | 29.47 (4.50) | 1,916 (190) | 29.49 (4.47) |
|  | **Males** | | | | | **Females** | | |
| **Variable** | **% MI** | **N (Missing)** | | **% CC** | | **% MI** | **N (Missing)** | **% CC** |
| Ethnicity |  | 1,101 (68) | |  | |  | 2,132 (0) |  |
| White | 96.0 | 1,058 | | 96.1 | | 96.0 | 1,865 | 96.0 |
| Non-white | 4.0 | 43 | | 3.9 | | 4.0 | 77 | 4.0 |
| Sex |  | 1,169 (0) | |  | |  | 2,132 (0) |  |
| Male | 100.0 | 1,169 | | 100.0 | | 0.0 | 0 | 0.0 |
| Female | 0.0 | 0 | | 0.0 | | 100.0 | 2,132 | 100.0 |
| Pregnancy size |  | 1,169 (0) | |  | |  | 2,131 (1) |  |
| Singleton | 98.3 | 1,149 | | 98.3 | | 97.5 | 2,078 | 97.5 |
| Multiple | 1.7 | 20 | | 1.7 | | 2.5 | 53 | 2.5 |
| Marital status |  | 1,110 (59) | |  | |  | 1,988 (144) |  |
| Married | 86.1 | 957 | | 86.2 | | 83.2 | 1,659 | 83.5 |
| Single | 9.3 | 102 | | 9.2 | | 12.9 | 252 | 12.7 |
| Widowed/separated/ divorced | 4.6 | 51 | | 4.6 | | 3.9 | 77 | 3.9 |
| Mother's education |  | 1,109 (60) | |  | |  | 1,967 (165) |  |
| O levels | 7.9 | 364 | | 32.8 | | 10.9 | 685 | 34.8 |
| A levels | 6.7 | 322 | | 29.0 | | 7.1 | 534 | 27.1 |
| Degree | 32.9 | 262 | | 23.6 | | 34.9 | 397 | 20.2 |
| CSE | 29.0 | 87 | | 7.8 | | 27.0 | 212 | 10.8 |
| Vocational | 23.6 | 74 | | 6.7 | | 20.1 | 139 | 7.1 |
| In poverty |  | 1,014 (155) | |  | |  | 1,771 (361) |  |
| No | 90.3 | 920 | | 90.7 | | 87.8 | 1,567 | 88.5 |
| Yes | 9.7 | 94 | | 9.3 | | 12.2 | 204 | 11.5 |
| Social class |  | 993 (176) | |  | |  | 1,714 (418) |  |
| Professional | 9.7 | 410 | | 41.3 | | 7.6 | 712 | 41.5 |
| Managerial and technical | 37.3 | 377 | | 38.0 | | 34.5 | 614 | 35.8 |
| Skilled non-manual | 41.6 | 98 | | 9.9 | | 42.5 | 139 | 8.1 |
| Skilled manual | 6.1 | 58 | | 5.8 | | 6.0 | 124 | 7.2 |
| Partly skilled | 4.7 | 45 | | 4.5 | | 7.6 | 99 | 5.8 |
| Unskilled | 0.7 | 5 | | 0.5 | | 1.8 | 26 | 1.5 |
| **Life periods** |  |  | |  | |  |  |  |
| Prenatal (age 18-32 weeks) |  | 1,005 (164) | |  | |  | 1,823 (309) |  |
| Not depressed | 83.8 | 796 | | 84.4 | | 81.9 | 1,502 | 82.4 |
| Depressed | 16.2 | 209 | | 15.6 | | 18.1 | 321 | 17.6 |
| Infancy (age 0-2 years) |  | 1,003 (166) | |  | |  | 1,755 (377) |  |
| Not depressed | 78.8 | 854 | | 79.2 | | 78.6 | 1,369 | 78.0 |
| Depressed | 21.2 | 149 | | 20.8 | | 21.4 | 386 | 22.0 |
| Early childhood (age 3-5 years) |  | 959 (210) | |  | |  | 1,705 (427) |  |
| Not depressed | 84.2 | 804 | | 85.1 | | 83.2 | 1,414 | 82.9 |
| Depressed | 15.8 | 155 | | 14.9 | | 16.8 | 291 | 17.1 |
| Middle childhood (age 6-8 years) |  | 971 (198) | |  | |  | 1,607 (525) |  |
| Not depressed | 83.0 | 796 | | 83.8 | | 81.1 | 1,299 | 80.8 |
| Depressed | 17.0 | 175 | | 16.2 | | 18.9 | 308 | 19.2 |
| Late childhood (age 9-12 years) |  | 795 (374) | |  | |  | 1,612 (520) |  |
| Not depressed | 81.1 | 667 | | 82.0 | | 79.4 | 1,287 | 79.8 |
| Depressed | 18.9 | 128 | | 18.0 | | 20.6 | 325 | 20.2 |
| Adolescence (age 19 years) |  | 640 (519) | |  | |  | 1,242 (890) |  |
| Not depressed | 82.0 | 573 | | 83.9 | | 80.7 | 1,042 | 83.9 |
| Depressed | 18.0 | 67 | | 16.1 | | 19.3 | 200 | 16.1 |
| A levels = Advanced levels (typically obtained at age 18); CC = complete cases; CSE = Certificate of Secondary Education (typically obtained at age 16, considered lower qualification than O-level); MI = multiple imputation; O-levels = Ordinary levels (typically obtained at age 16); SMFQ = Short Mood and Feelings Questionnaire; SD = standard deviation | | | | | | | | |

| **Table S5.** Prevalence of maternal depression at individual time points. | | | | |
| --- | --- | --- | --- | --- |
|  | **Males** | | **Females** | |
| **Age period** | **N** | **%** | **N** | **%** |
| 18 weeks gestation |  |  |  |  |
| Not depressed | 947 | 91.1 | 1,655 | 89.9 |
| Depressed | 93 | 8.9 | 185 | 10.1 |
| 32 weeks gestation |  |  |  |  |
| Not depressed | 960 | 89.2 | 1,688 | 88.2 |
| Depressed | 116 | 10.8 | 226 | 11.8 |
| 8 weeks |  |  |  |  |
| Not depressed | 1,002 | 92.6 | 1,757 | 92.3 |
| Depressed | 80 | 7.4 | 147 | 7.7 |
| 8 months |  |  |  |  |
| Not depressed | 1,007 | 92.9 | 1,768 | 92.7 |
| Depressed | 77 | 7.1 | 139 | 7.3 |
| 1 year and 9 months |  |  |  |  |
| Not depressed | 992 | 93.0 | 1,684 | 91.1 |
| Depressed | 75 | 7.0 | 164 | 8.9 |
| 2 years and 9 months |  |  |  |  |
| Not depressed | 938 | 89.8 | 1,631 | 90.1 |
| Depressed | 106 | 10.2 | 180 | 9.9 |
| 3 years and 11 months |  |  |  |  |
| Not depressed | 1,001 | 93.7 | 1,655 | 92.2 |
| Depressed | 67 | 6.3 | 140 | 7.8 |
| 5 years and 1 month |  |  |  |  |
| Not depressed | 917 | 90.4 | 1,564 | 88.9 |
| Depressed | 97 | 9.6 | 196 | 11.1 |
| 6 years and 1 month |  |  |  |  |
| Not depressed | 952 | 92.5 | 1,602 | 91.8 |
| Depressed | 77 | 7.5 | 143 | 8.2 |
| 8 years and 1 month |  |  |  |  |
| Not depressed | 912 | 90.6 | 1,534 | 88.2 |
| Depressed | 95 | 9.4 | 205 | 11.8 |
| 9 years 1 month |  |  |  |  |
| Not depressed | 914 | 88.3 | 1,545 | 87.2 |
| Depressed | 121 | 11.7 | 227 | 12.8 |
| 12 years 1 month |  |  |  |  |
| Not depressed | 913 | 90.9 | 1,520 | 90.2 |
| Depressed | 91 | 9.1 | 165 | 9.8 |
| 19 years |  |  |  |  |
| Not depressed | 667 | 83.9 | 1,042 | 83.9 |
| Depressed | 128 | 16.1 | 200 | 16.1 |

**Table S6.** Effect estimates (95% Cis) for selected lifecourse hypotheses

|  | **Males**  **n=1169** | | | **Females**  **n=2132** | | |
| --- | --- | --- | --- | --- | --- | --- |
| **Lifecourse hypothesis** | **Beta** | **95% CI** | **P value** | **Beta** | **95% CI** | **P value** |
| Accumulation | 0.113 | 0.073, 0.152 | <0.001 | 0.071 | 0.026, 0.119 | 0.002 |
| Sensitive period 6-8 years |  |  |  | 0.197 | -0.044, 0.344 | 0.049^a^ |

CI = Confidence Interval. ^a^The selective inference package is based on one-sided definition of p-value but 95% CIs are calculated based on a two-sided definition. Hence, it is possible to obtain p<0.05 but 95% CIs spanning 0.

Please note, the low correlation between predictors has led to selective inference being approximately equal to Bonferroni correction. Hence, statistics for the combination of hypotheses, which might not be calculable by selective inference, can be approximated through Bonferroni correction: for females, accumulation plus sensitive period 6-8 years gives Beta = 0.268, 95% CI: 0.034, 0.502, p= 0.015)
